# Gene expression profiles of esophageal squamous cell cancers in Hodgkin lymphoma survivors versus sporadic cases

**DOI:** 10.1371/journal.pone.0243178

**Published:** 2020-12-21

**Authors:** Berbel L. M. Ykema, Sanne J. M. Hoefnagel, Lisanne S. Rigter, Liudmila L. Kodach, Gerrit A. Meijer, Flora E. van Leeuwen, Hina N. Khan, Petur Snaebjornsson, Berthe M. P. Aleman, Annegien Broeks, Sybren L. Meijer, Kenneth K. Wang, Beatriz Carvalho, Kausilia K. Krishnadath, Monique E. van Leerdam, on behalf of GIOCA-OES

**Affiliations:** 1 Department of Gastroenterology and Hepatology, Netherlands Cancer Institute, Amsterdam, The Netherlands; 2 Department of Gastroenterology and Hepatology, Amsterdam UMC, Amsterdam, The Netherlands; 3 Center for Experimental and Molecular Medicine, Amsterdam UMC, Amsterdam, The Netherlands; 4 Department of Pathology, Netherlands Cancer Institute, Amsterdam, The Netherlands; 5 Department of Epidemiology, Netherlands Cancer Institute, Amsterdam, The Netherlands; 6 Department of Radiation Oncology, Netherlands Cancer Institute, Amsterdam, The Netherlands; 7 Core Facility Molecular Pathology and Biobanking, Division of Molecular Pathology, Netherlands Cancer Institute, Amsterdam, The Netherlands; 8 Department of Pathology, Amsterdam UMC, Amsterdam, The Netherlands; 9 Division of Gastroenterology and Hepatology, Mayo Clinic, Rochester, Minnesota, United States of America; 10 Department of Gastroenterology and Hepatology, Leiden University Medical Center, Leiden, The Netherlands; Ohio State University Wexner Medical Center, UNITED STATES

## Abstract

Hodgkin lymphoma (HL) survivors are at increased risk of developing second primary esophageal squamous cell cancer (ESCC). We aimed to gain insight in the driving events of ESCC in HL survivors (hESCC) by using RNA sequencing and NanoString profiling. Objectives were to investigate differences in RNA signaling between hESCC and sporadic ESCC (sESCC), and to look for early malignant changes in non-neoplastic esophageal tissue of HL survivors (hNN-tissue). We analyzed material of 26 hESCC cases, identified via the Dutch pathology registry (PALGA) and 17 sESCC cases from one academic institute and RNA sequencing data of 44 sESCC cases from TCGA. Gene expression profiles for the NanoString panel PanCancer IO 360 were obtained from 16/26 hESCC and four hNN-tissue, while non-neoplastic squamous tissue of four sporadic cases (sNN-tissue) served as reference profile. Hierarchical clustering, differential expression and pathway analyses were performed. Overall, the molecular profiles of hESCC and sESCC were similar. There was increased immune, HMGB1 and ILK signaling compared to sNN-tissue. The profiles of hNN-tissue were distinct from sNN-tissue, indicating early field effects in the esophagus of HL survivors. The BRCA1 pathway was upregulated in hESCC tissue, compared to hNN tissue. Analysis of expression profiles reveals overlap between hESCC and sESCC, and differences between hESCC and its surrounding hNN-tissue. Further research is required to validate our results and to investigate whether the changes observed in hNN-tissue are already detectable before development of hESCC. In the future, our findings could be used to improve hESCC patient management.

## Introduction

Hodgkin Lymphoma (HL) survivors treated with chemo- and/or radiotherapy have a 4–9 times increased risk of developing esophageal carcinoma (EC) compared to the general population [1–3]. Both second primary esophageal squamous cell carcinoma (ESCC) and esophageal adenocarcinoma (EAC) do occur in HL survivors, although the majority of ECs in HL survivors are ESCC (57%) [4].

Risk factors predicting which HL survivor will develop second primary ESCC (referred to as hESCC) are lacking. A dose-effect relationship has been shown between radiation to the esophagus and risk of EC [5]. Furthermore, procarbazine-containing chemotherapy is associated with an increased risk of second primary malignancies above the diaphragm [3]. A median interval time of 16 years between HL treatment and hESCC diagnosis has been reported [5].

Previous research has shown that the molecular basis of sporadic ESCC (sESCC) featured more resemblance with squamous cell carcinomas of other organs than with EAC [6–8]. The pathways involved in the development of sESCC include Wnt signaling, cell cycle regulation (mainly in G1/S transition control) and Notch pathways [6, 7, 9–12]. There has hardly been any reporting on the molecular basis of hESCC. Differences in the molecular profile of second primary solid malignancies in cancer survivors compared to sporadic solid malignancies have been described in other types of cancer, such as breast cancer, colorectal cancer and sarcomas. These differences retain to expression profiles, mismatch repair (MMR) and p53 status [13–17]. More insight in the molecular profiles and pathogenesis of hESCC and potential differences with sESCC might contribute to surveillance strategies and earlier diagnosis, preventive measures and future therapies [18].

We hypothesized that the molecular basis of hESCC differs from that of sESCC [5, 15, 19–23]. Therefore, we aimed to investigate the pathogenesis of these malignancies by comparing the molecular and expression profiles between the two groups. Treatment naïve non-neoplastic esophageal squamous tissues were taken as reference profiles. As a secondary aim, we compared non-neoplastic tissue of HL survivors (hNN-tissue), not treated with chemo- or radiotherapy for ESCC, with hESCC and non-neoplastic tissue of sporadic cases (sNN-tissue).

## Methods

### Study population and tissue samples

Twenty-six patients who were diagnosed with ESCC at least five years after the diagnosis of HL (referred to as hESCC) were selected based on a linkage request between PALGA (the nationwide network and registry of histo- and cytopathology in the Netherlands) and IKNL (Netherlands Comprehensive Cancer Organisation) (LZV1176), after approval by PALGA’s Scientific Council [24]. De-identified tissue samples and anonymized clinical data from the 18 hospitals participating to the PALGA registry were provided to the investigators by PALGA after pseudonymization. All tissue samples used were formalin-fixed paraffin-embedded (FFPE) from 2001–2015, and were either biopsy specimens obtained during gastroduodenoscopy (18/26, 69.2%) or surgical resection specimens (8/26, 30.8%). All patients were treated in Dutch hospitals and samples were collected in the 18 hospitals participating to the PALGA registry, but individualized information regarding location of sample collection was not available to the investigators. Material from all 26 patients was used for immunohistochemistry (IHC). A subset (n = 16) yielded sufficient material for RNA expression analyses, using NanoString technology [25]. Of these 16 samples, 10 (62.5%) were pretreatment specimens, one (6.2%) patient had received neo-adjuvant chemoradiotherapy before the surgical resection and for the remaining five (31.3%) patients no clinical information was available.

We also investigated non-neoplastic esophageal squamous FFPE tissue from four out of 26 hESCC cases, obtained before neoadjuvant chemo- or radiotherapy for hESCC (referred to as hNN-tissue). Treatment naïve non-neoplastic esophageal squamous FFPE material from four sESCC cases was used as reference for hESCC (referred to as sNN-tissue). These sNN-tissue samples were selected from the NKI hospital pathology archive.

For sporadic ESCC (referred to as sESCC), gender matched cases were recruited at the Amsterdam UMC (n = 32) for study purposes (referred to as sESCC AUMC). These patients had no history of other malignancy and were therefore considered sporadic. For validation purposes, data from sESCC patients from the TCGA database without prior malignancy (referred to as sESCC TCGA), was included (n = 44). Only patients from Western countries were included, to select for similar geographical background [26]. From these two series, the RNA sequencing profiles analyzed were from treatment-naïve neoplastic (AUMC n = 17 and TCGA n = 44) and non-neoplastic fresh-frozen biopsies from the esophagus (AUMC n = 10 and TCGA n = 11). Specifications of the AUMC and TCGA dataset are described in the S1 Appendix in S1 File [6].

### Ethical considerations

The protocol for retrieval of sESCC AUMC material was approved by the Medical Ethical Committee and/or AUMC-biobank committee of the AUMC (AMC 2013_241). This study received approval of the institutional research board of the Netherlands Cancer Institute (study number CFMPB307). Collection, storage and use of patient derived paraffine embedded tissue and data were performed in compliance with the “Code for Proper Secondary Use of Human Tissue in The Netherlands”, Dutch Federation of Biomedical Scientific Societies, the Netherlands and therefore no informed consent was obtained. For fresh frozen biopsies (AUMC RNA seq n = 17), patients provided written informed consent.

### Histopathological review

Pathology reports and FFPE tissues were obtained for histopathological revision. Hematoxylin & eosin (H&E) stained slides were analyzed according to standard protocol to confirm the diagnosis of ESCC and to determine differentiation grade and keratinization by an expert gastrointestinal pathologist (LK).

### Immunohistochemistry

IHC was performed on hESCC (n = 26) and sESCC from the AUMC (n = 26). IHC was performed for the MMR proteins according to standard protocols (as reported in the S1 File) for the Ventana automated immunostainer (MLH1 (Agilent / DAKO, Cat. # M3640), MSH2 (Roche / Ventana, Cat. # 8033684001), MSH6 (Epitomics, cat. # AC-0047EU) and PMS2 (Roche / Ventana, Cat. # 8033692001)). ESCC with absent staining of one or more MMR proteins were considered MMR-deficient. IHC was also performed for p53 (DO-7 antibody, DAKO, Agilent Technologies, Santa Clara, CA). Diffuse, strong nuclear staining (≥ 70%) or complete loss of staining were interpreted as aberrant p53 expression, indicating p53 mutation.

### NanoString for hESCC and hNN-tissue and sNN-tissue

The Allprep kit (Qiagen, United States) was used to isolate RNA from FFPE tissue of hESCC (n = 16), corresponding hNN-tissue not treated with chemo- and/or radiotherapy for ESCC (n = 4), and treatment naïve sNN-tissue (n = 4) according to protocol. Concentrations and purities were measured using Bioanalyzer (Agilent, Santa Clara, United States). Two runs with 12 samples were performed on the NanoString nCounterTM system (NanoString Technologies, Seattle, Washington, USA), using the PanCancer 360 IO panel (S1 Table in S1 File) [27]. mRNA levels were quantified according to NanoString’s instructions. Quality control was performed with the nSolver 4.0 software. Raw Transcript counts were analyzed for differential expression in the R language and environment for statistical computing. The NanoString panel was selected as described in S2 Appendix in S1 File. Heatmaps were generated as described in S3 Appendix in S1 File [28–30].

### RNA sequencing of sESCC

RNA was isolated using the Qiagen RNeasy kit according protocol from the fresh frozen samples including 17/32 cases of sESCC AUMC and 10 sNN-tissue, which served as a reference. The RNA served as input for RNA sequencing, which generated profiling of >15.000 genes. A quality control of RNA integrity was performed using the Agilent 2100 bioanalyzer (Agilent, Santa Clara, USA). RNA sequencing data generation and preparation was performed as described (S4 Appendix in S1 File). RNA sequencing data of the TCGA database of neoplastic (n = 44) and sNN-tissue (n = 11) was evaluated as described in S4 Appendix in S1 File.

### Differential expression analysis, gene set enrichment, ingenuity pathway analysis and statistical analyses

The sESCC AUMC (n = 17) and TCGA (n = 44) data series were analyzed using the Bioconductor package DESeq2 (version 1.14.1) [31]. All protein coding genes were compared. Adjusted p-values for differentially expressed genes were computed, after selecting the set of genes represented by the NanoString panel. Differential expression analyses on the Nanostring profiles were performed by using the glm.LRT function from the NanoStringDiff package [28, 29]. For this analysis, data obtained from the FFPE samples from hNN-tissue (n = 4) were used as comparison and data from sNN-tissue (n = 4) were used as the reference data. Batch effect corrections were included in the model.

Genes with an adjusted p-value of <0.05 (Benjamini and Hochberg) [32], were considered to be significantly differentially expressed with the same reference. The differentially expressed genes for the different datasets served as input for the Ingenuity Pathway Analysis tool (IPA, Qiagen, Hilden, Germany), to determine which gene sets were predicted to be activated and inactivated. Pathways with a -log p-value of 1.3 or higher and an absolute z-score of 2.0 or higher, were considered to be significantly differentially (in)activated.

IBM SPSS V.22.0 database software was used to analyze the clinical data. The χ^2^ tests, Fisher’s exact tests or Wilcoxon signed rank test were used to analyze categorical or continuous paired data. The significance level was defined as two-sides p<0.05.

## Results

### Clinical characteristics of hESCC and sESCC

**Fig 1:**
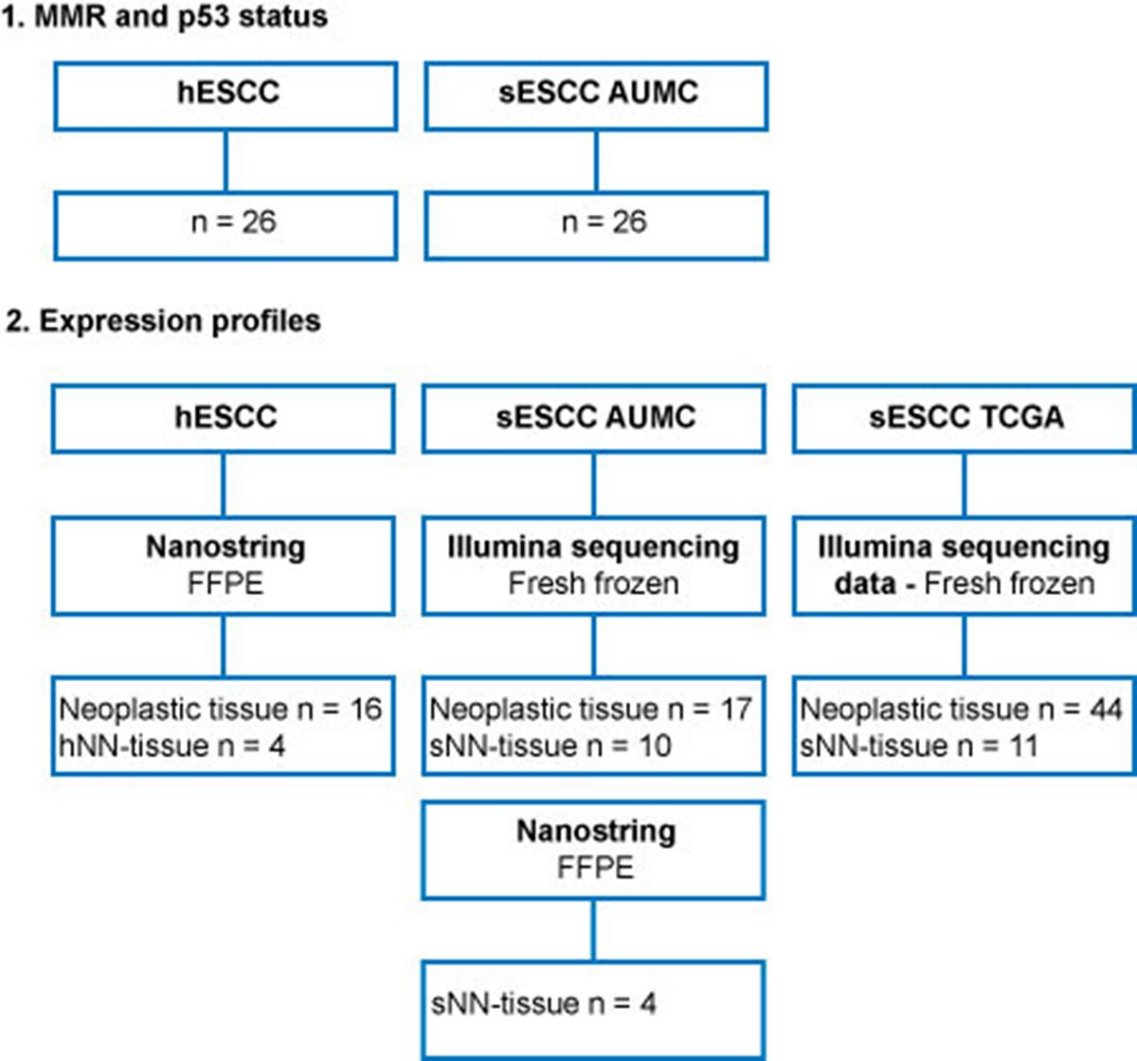
Overview of tissue samples for this study, mentioning the tissue samples used for MMR and p53 status and expression profiles separately. ESCC in HL survivors (hESCC) will be compared to sporadic ESCC (sESCC). For each group (HL survivors, AUMC and TCGA), non-neoplastic esophageal squamous tissue analyzed using the same platform (NanoString, Illumina sequencing), will be used as a reference for the analysis of the expression profiles of tumor tissue. This will be treatment naïve NN-tissue of sporadic cases (sNN-tissue) for the NanoString profiles and the Illumina sequencing profiles of AUMC and TCGA. NN-tissue of HL survivors (hNN-tissue), which had not been treated for ESCC with chemo- or radio-therapy, will also be analyzed by NanoString, as a comparator group.

A total of 26 HL survivors (50% male), diagnosed with HL between 1967 and 2007, were included. The median age at HL diagnosis was 36.5 years (range 20–76 years). HL treatment was variable over time and consisted of procarbazine-based chemotherapy and/or mantle-field irradiation or mediastinal irradiation [3]. The median interval between HL and hESCC diagnosis was 13 years (range 6–44 years, S2 Table in S1 File). The median age of the hESCC cases used for expression profile analysis was 55 years (range 33–85 years), which was comparable to the age of sESCC from the TCGA database (median 55.5 years; range 44–84 years), but significantly lower compared to the age at diagnosis in the sESCC AUMC cases (median 67 years; range 56–77 years). The percentage of males diagnosed with hESCC was comparable to sESCC AUMC, but significantly higher in the sESCC TCGA series. No differences were found for tumor location and histological grade. An overview of all cases is shown in Fig 1. All baseline characteristics of hESCC and sESCC series used for analysis of expression profiles are described in Table 1 and for MMR and p53 status in S2 Table in S1 File.

**Table 1:** Baseline characteristics RNA expression series of ESCC in Hodgkin lymphoma (HL) survivors (hESCC), sporadic ESCC (sESCC) of Amsterdam University Medical Center (AUMC) and sESCC of TCGA.

	hESCC n = 16	sESCC AUMC n = 17	sESCC TCGA n = 44	p-value
*HL treatment*		Not applicable	Not applicable	
Radiotherapy	2 (15.3%)			
Chemotherapy	1 (7.7%)			
Chemoradiotherapy	10 (77.0%)			
Unknown	3			
*Radiotherapy*				
Mantlefield	8 (of 12, 3 unknown, 1 not)			
*Age at diagnosis ESCC in years (median)*	55.0 (range 33–85)	67.0 (range 56–77)	55.5 (range 44–84)	0.011
*Year of diagnosis ESCC (median)*	2009 (range 1992–2015)	2013 (range 2012–2017)	2012 (range 2001–2013)	<0.001
*Sex*				0.065
Male	9 (56.3%)	8 (47%)	35 (79.5%)	
*Material*				
Tumor				
Treatment-naïve (No neoadjuvant chemo- and/or radiotherapy for ESCC)	10 (62.5%)	17 (100%)	44 (100%)	
Neoadjuvant chemo- and/or radiotherapy	1 (6.2%)			
No clinical information	5 (31.3%)			
Non-neoplastic tissue (No neoadjuvant chemo- and/or radiotherapy for ESCC)	4 (100%)	10 (100%)	11 (100%)	
*Tumor location*				0.128
Proximal	2 (16.7%)	3 (17.6%)	2 (5.3%)	
Middle	4 (33.3%)	6 (35.3%)	11 (28.9%)	
Distal	5 (41.7%)	6 (35.3%)	24 (63.2%)	
Junction/cardia	1 (8.3%)	2 (11.8%)	1 (2.6%)	
Missing	4	0	6	
*Histological grade*				0.139
Good	6 (37.5%)	1 (6.3%)	10 (26.3%)	
Moderate	10 (62.5%)	14 (87.4%)	20 (52.6%)	
Poor	0	1 (6.3%)	8 (21.1%)	
Missing	-	1	6	

### MMR and p53 status in hESCC and sESCC

MMR deficiency was detected in one out of 26 hESCC (4.0%) with loss of PMS2/MLH1 protein staining (no MLH1 methylation) and in none of the sESCC AUMC. There was no difference in p53 expression between hESCC and sESCC AUMC (S2 Table in S1 File).

### Signaling pathways involvement in hESCC and in sESCC using sNN-tissue as a reference

**Fig 2:**
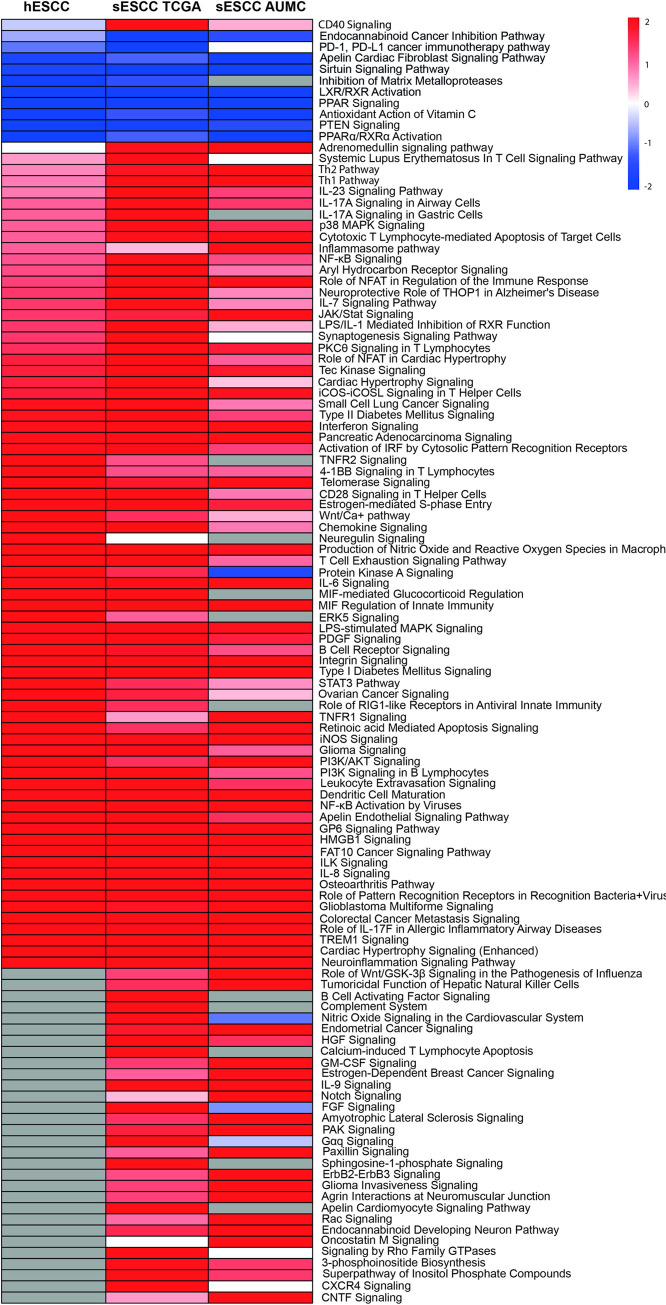
Heatmap of differentially activated pathways when comparing tumor tissue of the three different series versus treatment naïve non-neoplastic tissue from sporadic cancers (sNN-tissue) reference. Of the Pancancer IO 360 Gene expression Panel, 242 genes were differentially expressed between hESCC and the non-neoplastic squamous control tissues (sNN); 610 between the sESCC AUMC and the sNN and 325 between the sESCC TCGA and sNN tissues. These differentially expressed genes were used as input to perform the IPA analysis. In general, similar pathways are (in)activated in the three different patient series (HL survivors, AUMC, TCGA) comparing neoplastic and sNN-tissue. Deactivated pathways in all three different series are PPAR (peroxisome proliferator-activated receptor) signaling and LXR/RXR activation. Activated pathways are immune-related pathways, HMGB1 signaling and colorectal cancer metastasis signaling and ILK signaling.

To investigate which signaling pathways were involved and if there was a difference in signaling between hESCC and sESCC, we performed IPA. Treatment naïve sNN-tissue for the corresponding series was used as a reference. We found 242, 610 and 325 genes from the PanCancer IO 360 Gene Expression Panel to be differentially expressed between neoplastic tissue and sNN-tissue, for the hESCC, sESCC AUMC and sESCC TCGA series, respectively. IPA showed up- or down-regulation for several pathways in the ESCC series (Fig 2).

Overall a similar pattern of pathway up- or downregulation was detected for hESCC, sESCC AUMC and sESCC TCGA, and thus without a specific pattern for hESCC (Fig 2).

The two pathways that were significantly deactivated in hESCC and sESCC were PPAR (peroxisome proliferator-activated receptor) signaling and LXR/RXR activation, which are involved in the regulation of the lipid metabolism [33, 34]. Seventeen pathways were significantly activated in both hESCC and sESCC. Among these pathways many were immune related pathways (e.g. role of pattern recognition receptors in recognition of bacteria and viruses, IL-8 signaling, IL-6 signaling, dendritic cell maturation and MIF regulation of the innate immunity). HMGB1 signaling, associated with inflammatory responses and tumor metastasis, was also activated. Other cancer progression pathways that were activated included colorectal cancer metastasis signaling and ILK signaling, which is implicated in connecting integrins to the cytoskeleton [35].

### Principal component analysis and unsupervised clustering of hESCC, hNN-tissue and sNN-tissue

By NanoString analysis, the expression of a subset of 770 genes was investigated in the hESCC. To investigate for precursor events, we also investigated hNN-tissue (n = 4). The NanoString profiles from sNN-tissue (n = 4) were used as the reference.

**Fig 3:**
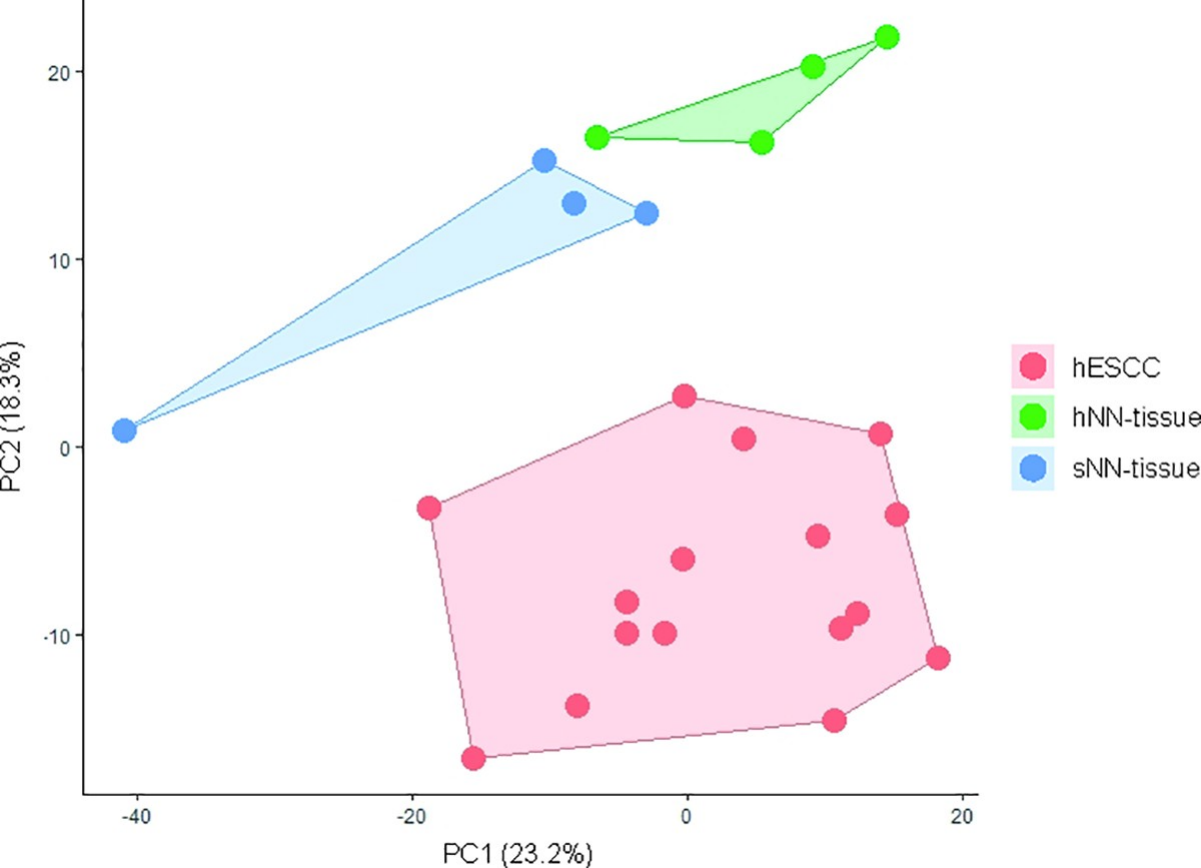
Principal component analysis (PCA) plot showing principal component 1 (x-axis) and 2 (y-axis) scores for the different sample types analysed with NanoString. Non-neoplastic tissue from HL survivors (hNN-tissue) is distinguishable from non-neoplastic tissue from sporadic ESCC cases (sNN-tissue) and ESCC in HL survivors (hESCC) on this PCA plot. PCA plots from sporadic ESCC are shown in a separate figure, since RNA sequencing was performed, which is not comparable to NanoString profiling (S2 Fig in S1 File).

Principal Component Analysis (PCA) was performed to provide an overview of clustering of the NanoString profiles obtained from the FFPE samples and yielded three distinct subgroups, including a group of hESCC, a second group consisting of the hNN-tissue, and a third group representing the sNN-tissue (Fig 3).

**Fig 4:**
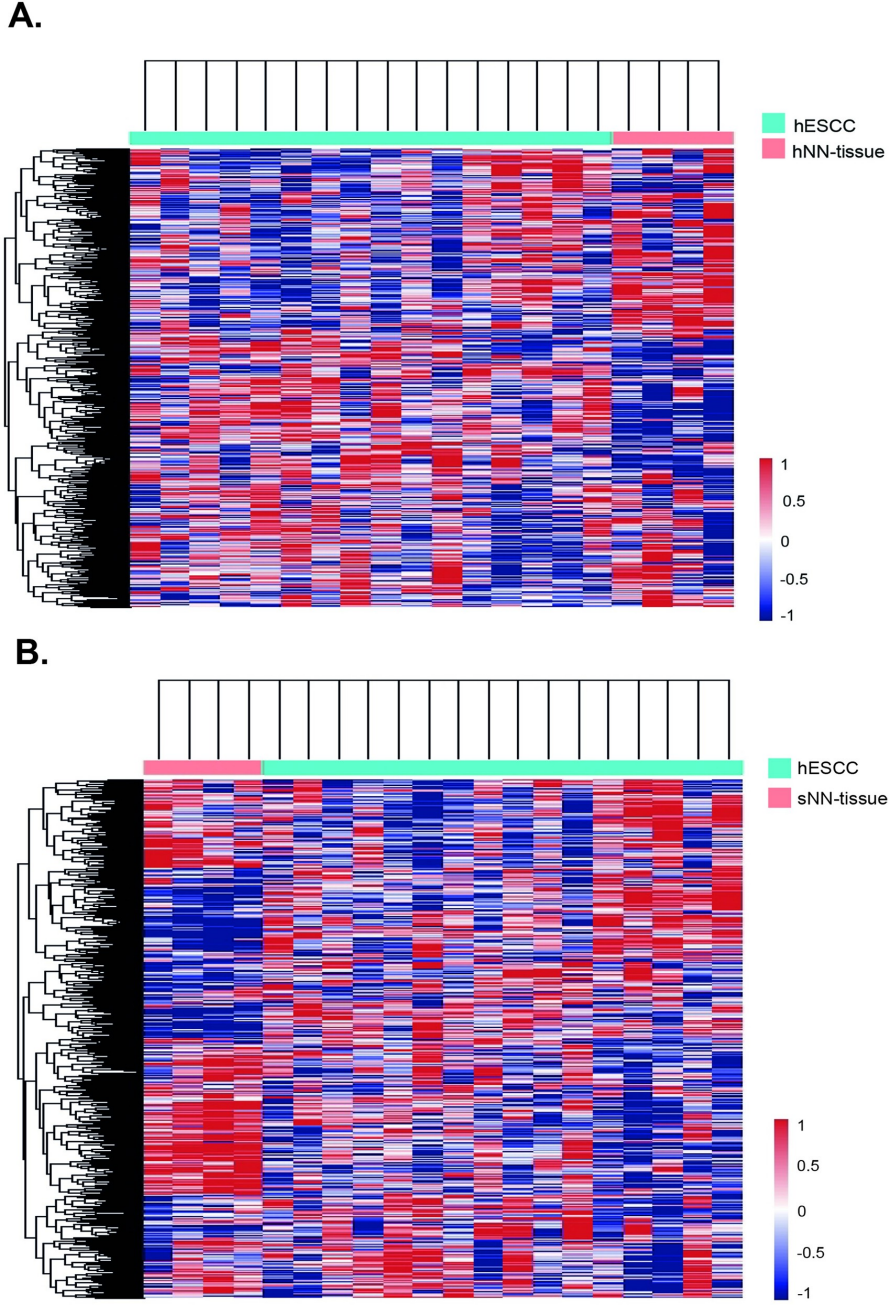
Heatmaps showing hierarchical cluster analyses results when applied to NanoString profiles. Cluster analyses on one ESCC in HL survivors (hESCC) and non-neoplastic tissue from HL survivors (hNN-tissue) (Fig 4A) and 2. hESCC and sNN-tissue (Fig 4B) result in separate tumor and non-neoplastic tissue groups.

Based on this finding we subsequently performed hierarchical unsupervised clustering, which results in a clear separation of hESCC versus hNN-tissue (Fig 4A) and hESCC versus sNN-tissue (Fig 4B). The cluster membership did not show a correlation between sample pairs of hNN-tissue and hESCC from the same HL patient (S1 Fig in S1 File). Two separate PCA plots show that sESCC is distinguishable from sNN-tissue and TCGA data generated by RNA sequencing (S5 Appendix and S2 Fig in S1 File).

### Differential expression analysis and ingenuity pathway analysis of hESCC compared to hNN-tissue

From the NanoString set, 191 genes were differentially expressed between the hESCC and hNN-tissue. In order to identify signaling pathways that are differentially expressed between the hESCC and hNN-tissue, IPA was performed using these genes as input.

Fifty-one pathways were significantly down-regulated in hESCC compared to the hNN-tissue. These included pathways important for different immune cell types from leucopoietic, myeloid and lymphoid progenitor cells, such as dendritic cells, macrophages and T- and B-lymphocytes.

**Fig 5:**
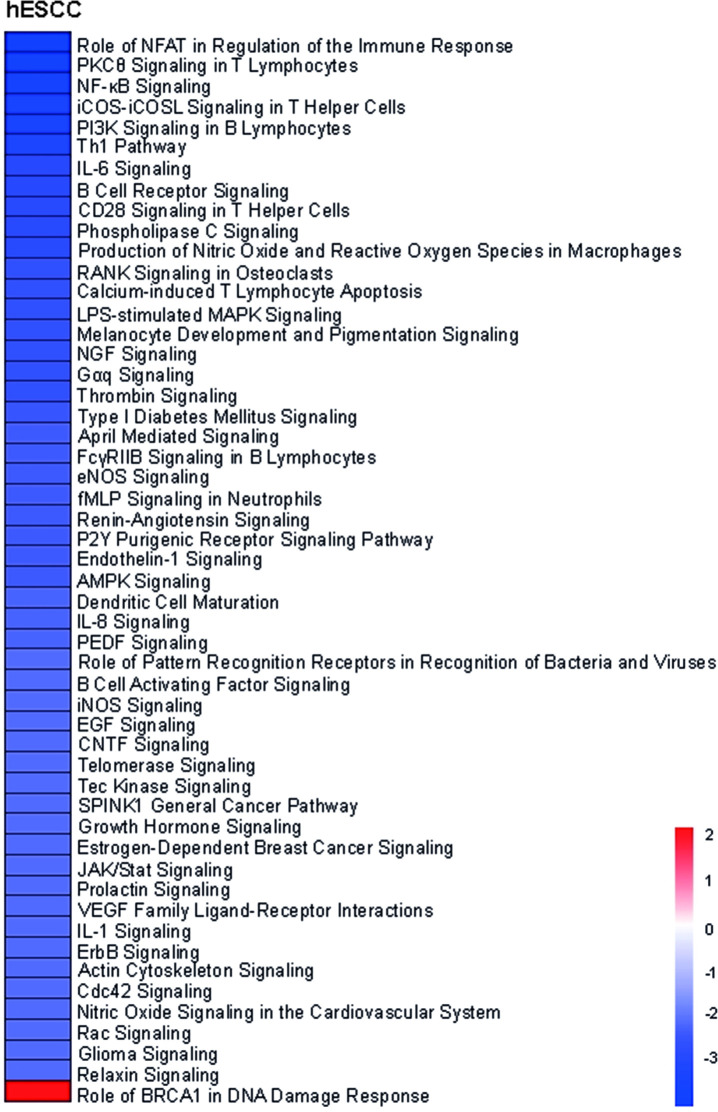
Heatmap of differentially activated pathways when comparing ESCC in HL survivors (hESCC) versus non-neoplastic tissue from HL survivors (hNN-tissue). The z-scores of the significant activated/deactivated pathways estimated by ingenuity pathway analyses are visualized. The “Role of BRCA1 in DNA damage response” is the only significantly activated pathway in hESCC.

We identified the “Role of BRCA1 in DNA damage response” as the only pathway that was significantly more activated in hESCC compared to the hNN-tissue (Fig 5).

### Genes and signaling pathways that are uniquely expressed in hESCC and hNN-tissue compared to sNN-tissue

We also identified those genes that were uniquely expressed in hESCC and hNN-tissue, compared to SNN-tissue. In the first comparison between hNN-tissue and sNN-tissue, 238 genes were differentially expressed. IPA analysis indicated that these genes are related to five pathways which are down-regulated and 102 pathways that are up-regulated in hNN-tissue, including colorectal cancer metastasis signaling (S3 Fig in S1 File). In the second comparison between hESCC and sNN-tissue, 242 genes were differentially expressed, as indicated in the caption of Fig 2. To gain more insight in which genes and signaling pathways might be involved early on in the development of hESCC, we identified genes that were similarly expressed in the hESCC and the hNN-tissue, but differentially expressed between hESCC and sNN-tissue (S4 Fig in S1 File).

**Fig 6:**
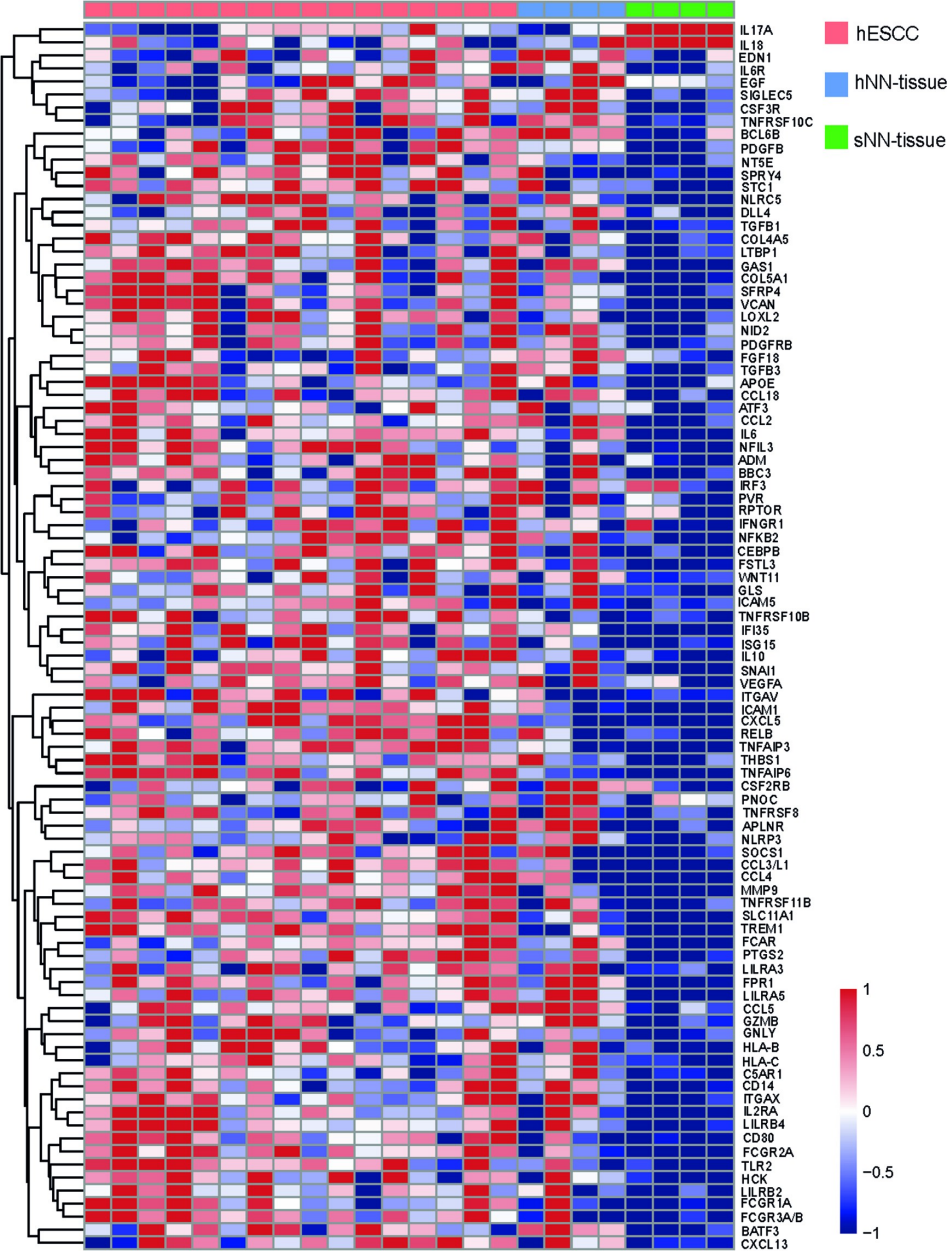
Heatmap showing the 94 differentially expressed genes with similar expression in non-neoplastic tissue from HL survivors (hNN-tissue) and ESCC from HL survivors (hESCC), when compared to non-neoplastic tissue from sporadic ESCC (sNN-tissue).

Through this analysis, we identified 94 genes (S4 Fig in S1 File), which were similarly expressed in both hESCC and the hNN-tissue (92 genes with similar up regulation and two genes with similar down-regulation, Fig 6). These genes had opposite expression values in sNN-tissue. From these 94 genes, 7 genes (ATF3, BATF3, CEBPB, IRF3, NFKB2, NFIL3, RELB) were listed with description in S4 Table in S1 File, based on their GO annotation as transcription factor.

## Discussion

In this study comparing molecular profiles from hESCC and sESCC, we have shown that both p53 and MMR status as well as RNA expression profiles are similar. To our knowledge, this is the first study that compares hESCC to sESCC at the RNA level. Moreover, this study includes the largest number of hESCC (n = 26) analyzed so far [20].

Despite the fact that several studies have suggested a potential role of MMR deficiency in second primary malignancies [15, 20, 36, 37], we detected only one MMR deficient hESCC in our series. This is in line with an earlier smaller study on second primary ESCCs, in which therapy-related and sporadic ESCC were not different with respect to microsatellite instability (MSI) events [20].

Similar pathways at the RNA expression level are (de)activated in hESCC and sESCC. This suggests similar molecular cancer mechanisms at the RNA level between the two groups, rather than a distinguishable and specific signature for hESCC. Activation of Wnt signaling and Notch pathways, earlier shown to be involved in development of ESCC, are not clearly different between hESCC and sESCC in our data [6, 7, 9–12]. These data differ from previous results, which concluded that the RNA expression profiles of second primary radiation-induced breast cancer and sarcomas were different compared to sporadic forms [13, 14]. On the other hand, an overall similar frequency of loss of heterozygosity has been reported in second primary EC (including ESCC and EAC) in HL and breast cancer survivors compared to sporadic EC [20].

We also analyzed the neoplastic tissue in comparison to its neighboring hNN-tissue that had not been treated for ESCC with chemo- and/or radiotherapy. From this analysis we conclude that immune pathway signaling associated with myeloid and lymphoid progenitor cells are lower in hESCC compared to hNN-tissue. Additional studies to investigate possible underlying mechanisms are needed to explain these differences, while larger series are required to evaluate their implication for HL patient management.

The only activated pathway that we identified when we analyzed the hESCC versus the hNN-tissue includes genes involved in the “Role of BRCA1 in DNA damage response” (downregulation of MLH1, upregulation of BRCA1, BRIP1 and RAD51). Thus, the tumor suppressor gene BRCA1, upregulated in hESCC, might play a role in the DNA damage response in hESCC. In a physiological state, BRCA1 functions as a tumor suppressor by forming a complex with DNA damage repair proteins including MSH2, MSH6, MLH1, ATM and BLM, and plays an important role in recognizing and repairing DNA damage [38]. Possible significance of this tumor suppressing pathway in hESCC will be investigated in future research.

As a secondary aim we investigated hESCC, hNN-tissue and sNN-tissue, and we show distinct patterns between hNN-tissue and sNN-tissue and a remarkable overlap between hESCC and hNN-tissue. This is in concordance with post-treatment mutational signatures, which have been reported in non-neoplastic tissue after different anti-cancer treatments [16]. We find 94 genes in hNN-tissue with a similar expression pattern as in hESCC (92 genes up-regulated and 2 down-regulated). These genes seem to be characteristic for the carcinogenic process in HL survivors and several of these genes could be induced by the treatment for HL consisting of chemo- and/or radiotherapy. Therefore, these early changes could potentially serve as unique prognostic factors and changes associated with early onset of disease. Therefore, these genes require further investigation in, for instance, HL survivors that do not developed ESCC, for further validation of their potential as prognostic biomarkers. Of note, the sample size of pre-treatment hNN-tissue and sNN-tissue was low in both groups (n = 4). The availability of pre-treatment material specimens needed to obtain non-neoplastic tissue in HL survivors with a second primary ESCC before neo-adjuvant therapy for ESCC was very limited. However, further research on more samples could confirm our findings and provide possible explanations, such as field cancerization. Field cancerization is a process in which large areas of cells are affected by carcinogenic alterations at the genetic and epigenetic level [39, 40]. This may imply that hESCC and altered gene expression in hNN-tissue are therapy-related, possibly with other etiological changes also being involved.

Several limitations to this study result from the fact that hESCC is a rare disease and that access to patient material and data is therefore limited. No information was available about treatment of HL of 10/26 hESCC. Dutch HL treatment guidelines at the time of HL diagnoses between the period of 1967 and 2007 varied, including the chemo- and/or radiotherapy regimens as described previously [3].

A second limitation is that for RNA profiling, RNA derived from samples stored in two different ways and two different techniques (RNA sequencing and NanoString) were used, however complementarity between the two techniques has been shown [41]. As hESCC material was collected for diagnostic purposes, the only accessible (rest) material was FFPE material. RNA was therefore more degraded and could be analyzed by NanoString, but not by RNA sequencing. Therefore whole transcriptome analysis was not possible, but only NanoString analysis of a subset of genes, and we may have missed critical gene expression signatures in hESCC. In contrast, for sESCC, RNA isolated from fresh frozen tissue, collected for research purposes, served as input for RNA sequencing.

## Conclusions

This is a preliminary step in investigating underlying molecular mechanisms in hESCC. We demonstrated striking similarities between the gene expression profiles of hESCC and sESCC. Further research is necessary to evaluate whether observed changes in the hNN-tissue are already detectable before development of ESCC, which could improve surveillance strategies and treatment options for this specific group of patients.

## Supporting information

S1 File (DOCX)

S1 Data NanoString counts exported from nSolver. (TXT)

S2 Data Annotation file NanoString. (XLSX)
