# Supplementary material for: Gene expression profiles of esophageal squamous cell cancers in Hodgkin lymphoma survivors versus sporadic cases
Source: PLoS One. 2020 Dec 21;15(12):e0243178. doi: 10.1371/journal.pone.0243178 (PMC7751872; doi:10.1371/journal.pone.0243178)
Supplement: S1 File — (DOCX) [file pone.0243178.s001.docx]

*Gene expression profiles of esophageal squamous cell cancers in Hodgkin lymphoma survivors versus sporadic cases*

**S1 Appendix – Methods AUMC and TCGA cohorts**

**AUMC patient cohort**

Patients were only included after informed consent. These patients were diagnosed with ESCC between 2005 and 2017 and material was available in the Biobank of the AUMC. Biopsies were collected in formalin or in RNA later (for storage at -80 degrees) during endoscopy, which was performed for diagnostic and tumor staging purposes prior to treatment. FFPE tissues were analyzed by IHC for 26 cases (one to one matched for sex with the HL survivors cases). Of 17 patients, frozen material was available for RNA sequencing (referred to as sESCC AUMC). As a reference, non-neoplastic tissue from EAC cases (referred as sNN-tissue) was used (n=10).

**TCGA patient cohort**

TCGA data of sporadic ESCC were used to validate the data. As reference data, we used non-neoplastic tissue from patients with sporadic EAC (and one with EC but no specification of subtype, n=11) from the TCGA database (sNN-tissue). All RNA sequencing profiles generated by the TCGA were from treatment-naïve, fresh-frozen tissues [2].

**S2 Appendix – Immunohistochemistry protocol Mismatch repair proteins**

Immunohistochemistry of the FFPE tumor samples was performed on a BenchMark Ultra autostainer (Ventana Medical Systems). Briefly, paraffin sections were cut at 3 um, heated at 75°C for 28 minutes and deparaffinized in the instrument with EZ prep solution (Ventana Medical Systems). Heat-induced antigen retrieval was carried out using Cell Conditioning 1 (CC1, Ventana Medical Systems) for 32 minutes at 950C (MLH1, MSH2 and MSH6), or 72 minutes at 950C (PMS2).

MLH1 was detected using clone ES05 (1/20 dilution, 32 minutes at 370C, Agilent / DAKO), MSH2 using clone G219-1129 (Ready-to-Use, 12 minutes at 370C, Roche / Ventana), MSH6 clone EP49 (1/50 dilution, 32 minutes at 370C, Epitomics) and PMS2 using clone A16-4 (ready-to-Use, 32 minutes at 370C, Roche / Ventana). For MLH1 and PMS2 signal amplification was applied using the Optiview Amplification Kit (4 minutes, Ventana Medical Systems). Bound antibody was detected using the OptiView DAB Detection Kit (Ventana Medical Systems).

Slides were counterstained with Hematoxylin and Bluing Reagent (Ventana Medical Systems).

**S3 Appendix – Methods selection of Nanostring panel**

**Selection of Nanostring panel**

To select a panel of genes to address differences between sESCC and hESCC, we first performed differential expression analyses using the DESeq function from DESeq2 comparing sporadic ESCC to sNN-tissue in the AUMC and TCGA datasets in separate analysis. Next, we defined which number of differentially expressed genes (according to adjusted p-values) were represented by different Nanostring panels important in cancer and immunology. The Nanostring panel PanCancer 360 IO representing the most differentially expressed genes in the AUMC dataset was used to perform transcriptome profiling in HL survivors.

**S4 Appendix – Methods Heatmaps of the Nanostring profiles**

**Heatmaps of the Nanostring profiles**

Variance stabilizing normalization of the transcript counts using the NanoStringNorm package was performed [3]. The normalized profiles were used for z-score calculation and visualization in heatmaps [4]. Samples were depicted by columns, and genes were depicted by rows. Both columns and rows were clustered unsupervised based on the complete linkage method on Maximum distances, to find similar clusters of respectively samples and genes.

**S5 Appendix - Quality control of RNA integrity for RNA sequencing data of sESCC**

**Quality control of RNA integrity for RNA sequencing data of sESCC**

Sample preparation of RNA sequencing data of 17 tumor tissues of sESCC AUMC and 10 sNN-tissue AUMC was performed using the NEBNext Ultra Directional RNA library Prep Kit for Illumina according to manufacturer’s protocol (NEB #E7420S/L, New England BioLabs Inc, Ipswich, USA). Clustering and cDNA sequencing with 75 bases single end run using the Illumina NextSeq500 was performed according to manufacturer’s protocols. Image analysis, base calling, and quality check was performed with the Illumina data analysis pipeline RTA v2.4.11 and Bcl2fastq v2.17. The reads were mapped to the Genome Reference Consortium Human genome build 38 patch release 7 (GRCh38.p7). Alignment was performed using Tophat2 version 2.1.1 with default parameters.27 The frequency of how often a read was mapped on a transcript was determined using featureCounts v1.5.0-p1.28 The counts were saved to count files, which served as input into the R language and environment for statistical computing.

**Supporting Results**

**S6 Appendix. Results Principal Component Analysis between sESCC and sNN-tissue**

**Principal Component Analysis between sESCC and sNN-tissue**

Expression of the 770 genes from the Nanostring Panel was obtained from the Illumina RNA-sequencing data of the sESCC of the AUMC (n=17) and TCGA (n=44). RNA-sequencing data from frozen normal esophageal squamous biopsies was used as a reference (sNN-tissue). The data obtained from the Nanostring and from the RNA sequencing samples are plotted separately. These PCA plots again clearly separate the sESCC from the never treated normal squamous esophageal samples (sNN-tissue) (S2 Fig).

**Supporting Tables**

**S1 Table**. Pathways included in PanCancer 360 IO panel of Nanostring

| **Annotation** | **Pathways represented** |
| --- | --- |
| Release of Cancer Cell Antigens | Microsatellite Instability (MSI) |
|  | Double Strand Break Repair |
|  | Chromatin Modification/Epigenetics |
| Cancer Antigen Presentation | MHC Class-I/II genes |
|  | Non-MHC Antigen Presentation |
|  | Antigen Processing Machinery |
|  | Proteasome and Immunoproteosome |
|  | Cross-presenting Dendritic Cell Genes |
| T-cell Priming and Activation | Costimulatory Molecules |
| Immune Cell Localization to Tumors | Chemokines |
|  | Integrins |
|  | Selectins |
|  | Immune Cell Populations in Tumors |
| Stromal Factors | Extracellular Matrix Remodeling |
|  | Collagens |
|  | Angiogenesis |
|  | Metastasis |
| Recognition of Cancer Cells by T-cells | Immune checkpoints |
| Killing of Cancer Cells | Interferon Signaling |
|  | JAK-STAT1/2 Pathway |
|  | Cytolytic Activity |
|  | Phagocytosis |
| Myeloid Cell Activity | Inflammation |
|  | Fc-gamma Receptor Signaling |
| NK Cell Activity |  |
| Cell Cycle and Proliferation |  |
| Tumor-Intrinsic Factors | Apoptosis |
|  | Autophagy |
|  | Nutrient Depletion |
|  | Metastasis |
| Immunometabolism | Oxygen Sensing |
|  | Nutrient regulation |
| Common Signaling Pathways | Wnt |
|  | Hedgehog |
|  | TGF-beta |
|  | NF-kappaB |
|  | Notch |
|  | PI3K-Akt |
|  | RAS |
|  | MAPK |
| Internal Reference Genes |  |

**S2 Table**. Baseline characteristics for matched esophageal squamous cell cancer (ESCC) in Hodgkin lymphoma survivors (hESCC) and sporadic cases (sESCC) from the AUMC for immunohistochemistry

|  | **hESCC**  **n = 26** | **sESCC AUMC**  **n = 26** | **p-value by Wilcoxon rank test** |
| --- | --- | --- | --- |
| *History of oncology/HL*  HL age of diagnosis  Interval HL and ESCC  HL stage  I  II  III  IV  Unknown  HL treatment  Chemotherapy  Radiotherapy  Combination  Unknown | 36.5 (range 20 – 76)  13 (range 6 – 44)  3 (11.5%)  5 (19.3%)  2 (7.7%)  2 (7.7%)  14 (53.8%)  1 (3.8%)  2 (7.7%)  13 (50.0%)  10 (38.5%) | - -  - |  |
| *Median age at diagnosis ESCC* | 55 (range 33 - 85) | 67 (49 – 78) | 0.036 |
| *Median year of diagnosis ESCC* | 2006 (range 1990 - 2015) | 2012 (range 2005-2017) |  |
| *Sex*  Male | 13 (50.0%) | 13 (50.0%) | 1.00 |
| *Tumor location*  Proximal Middle Distal Junction  Unknown | 4 (15.4%)  6 (23.1%)  5 (19.2%)  2 (7.7%)  9 (34.6%) | 5 (19.2%)  10 (38.5%)  10 (38.5%)  1 (3.8%)  - | 0.010 |
| *Histological grade*  Differentiation grade  Good  Moderate  Poor  Unknown  Keratinization  Present  Not present  Unknown | 9 (34.6%)  13 (50.0%)  2 (7.7%)  2 (7.7%)  15 (57.7%)  9 (34.6%)  2 (7.7%) | 2 (7.7%)  21 (80.8%)  2 (7.7%)  1 (3.8%)  7 (26.9%)  13 (50.0%)  6 (23.1%) | 0.410  0.514 |
| *p53*  Negative  Wild-type  Overexpression  Unknown | 5 (19.2%)  2 (7.7%)  16 (61.6%)  3 (11.5%) | 7 (26.9%)  6 (23.1%)  11 (42.3%)  2 (7.7%) | 0.344 |
| *Loss of MMR protein staining*  No (MLH1+ PMS2+ MSH2+ MSH6+)  PMS2 – with abnormal MLH1  Unknown | 24 (92.3%)   1 (3.8%)  1 (3.8%) | 25 (96.2%)  -  1 (3.8%) | 0.317 |

**S3 Table.** Number of differentially expressed genes, resulting from comparison between ESCC and non-neoplastic tissue (sNN-tissue) in RNA sequencing datasets, represented by Nanostring panels.

| **Panel** | **AUMC** in panel/not in panel | **TCGA** in panel/not in panel |
| --- | --- | --- |
| PanCancer IO 360 | 601/9501 | 381/7838 |
| Breast Cancer 360 | 585/9517 | 446/7773 |
| PanCancer Progression | 577/9525 | 384/7835 |
| Human Myeloid Innate Immune V2 | 547/9555 | 356/7863 |
| Human AutoImmune Profiling | 531/9571 | 321/7898 |
| Human AutoImmune discovery | 513/9589 | 501/9606 |
| PanCancer Immune | 504/9598 | 315/7904 |
| PanCancer Pathways | 501/9601 | 398/7821 |
| Human Immunology V2 | 410/9692 | 549/9558 |
| Human Kinase | 340/9762 | 219/8000 |
| Human Inflammation V2 | 178/9924 | 107/8112 |

**S4 Table.** Shortlist of potential biomarkers with go_id annotation 0003700 (transcription factor) filtered from 94 genes with similar expression patterns in hESCC and hNN-tissue.

| **Gene name** | **Description** | **go_id** |
| --- | --- | --- |
| ATF3 | activating transcription factor 3 [Source:HGNC Symbol;Acc:HGNC:785] | GO:0003700 |
| BATF3 | basic leucine zipper ATF-like transcription factor 3 [Source:HGNC Symbol;Acc:HGNC:28915] | GO:0003700 |
| NFKB2 | nuclear factor kappa B subunit 2 [Source:HGNC Symbol;Acc:HGNC:7795] | GO:0003700 |
| RELB | RELB proto-oncogene, NF-kB subunit [Source:HGNC Symbol;Acc:HGNC:9956] | GO:0003700 |
| IRF3 | interferon regulatory factor 3 [Source:HGNC Symbol;Acc:HGNC:6118] | GO:0003700 |
| CEBPB | CCAAT enhancer binding protein beta [Source:HGNC Symbol;Acc:HGNC:1834] | GO:0003700 |
| NFIL3 | nuclear factor, interleukin 3 regulated [Source:HGNC Symbol;Acc:HGNC:7787] | GO:0003700 |

**Supporting Figures

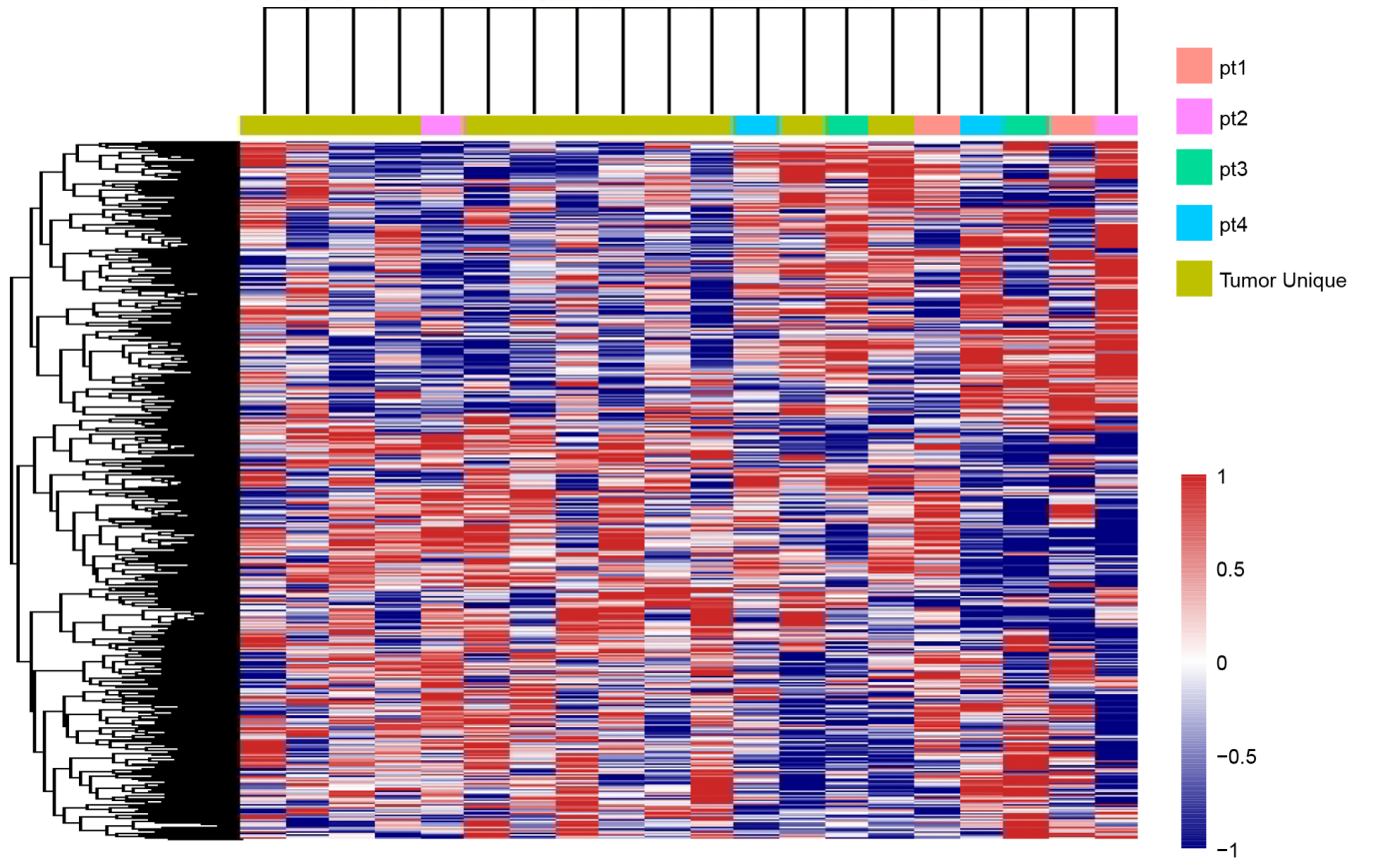
**

**S1 Fig.** Heatmap showing same clustered heatmap as in Fig 4A, but annotated for patient ID in case multiple tissues were derived from the same patient. The clustering of expression profiles does not lead to separation of sample pairs (non-neoplastic squamous tissue (hNN-tissue) and tumor tissue) derived from the same patient (this figure).

**
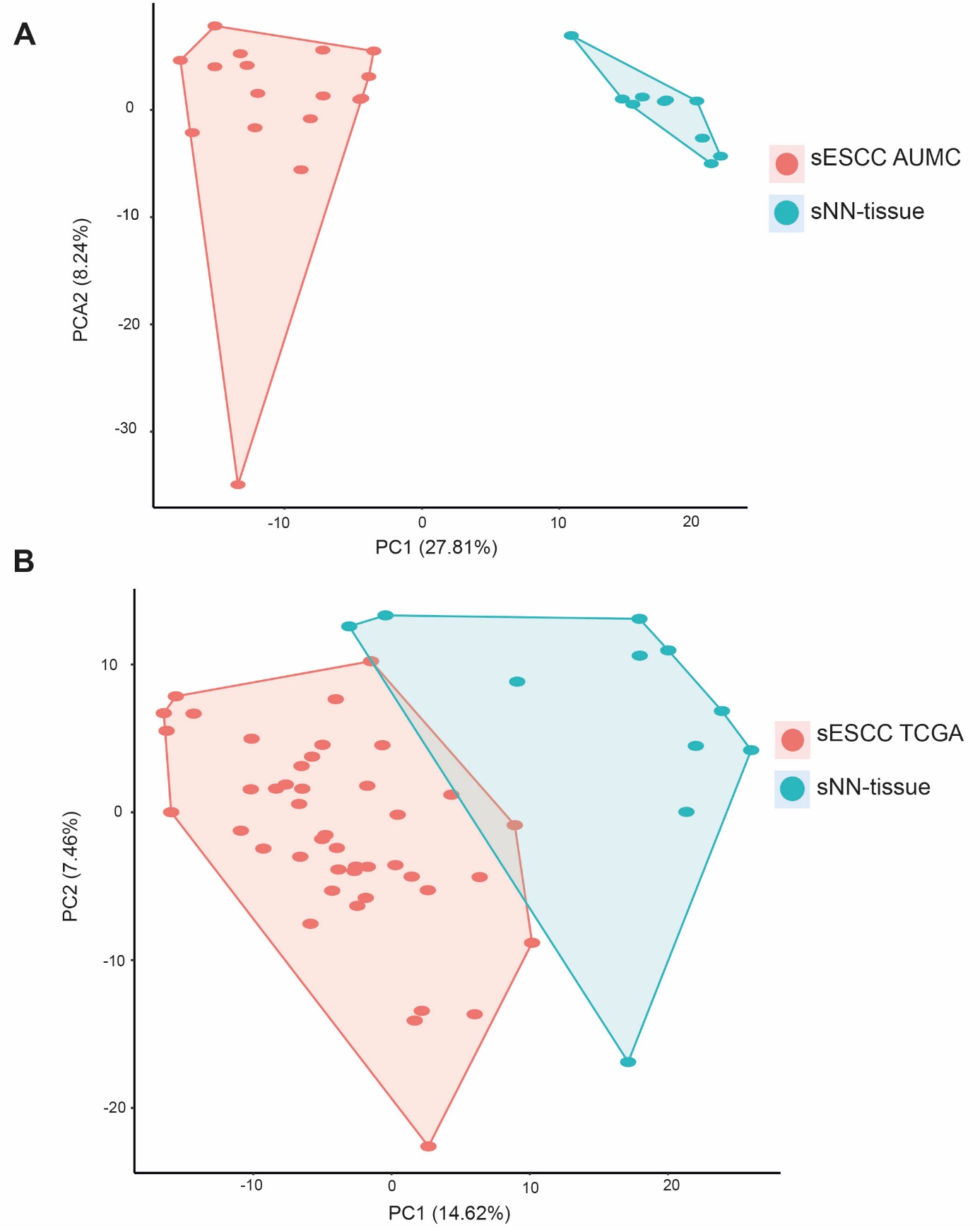
**

**S2 Fig.** Principal component analysis (PCA) plots showing principal component 1 (x-axis) and 2 (y-axis) scores for the different sample type profiles generated by RNA sequencing. The same genes as analysed by the Nanostring PanCancer IO 360 panel were used as input for the PCA after vsd normalization. In the sporadic ESCC (sESCC) AUMC database (A) and the sESCC TCGA database (B), non-neoplastic tissue from sporadic cases (sNN-tissue) is distinguishable from ESCC from sESCC cases (ESCC of sporadic cases).

**
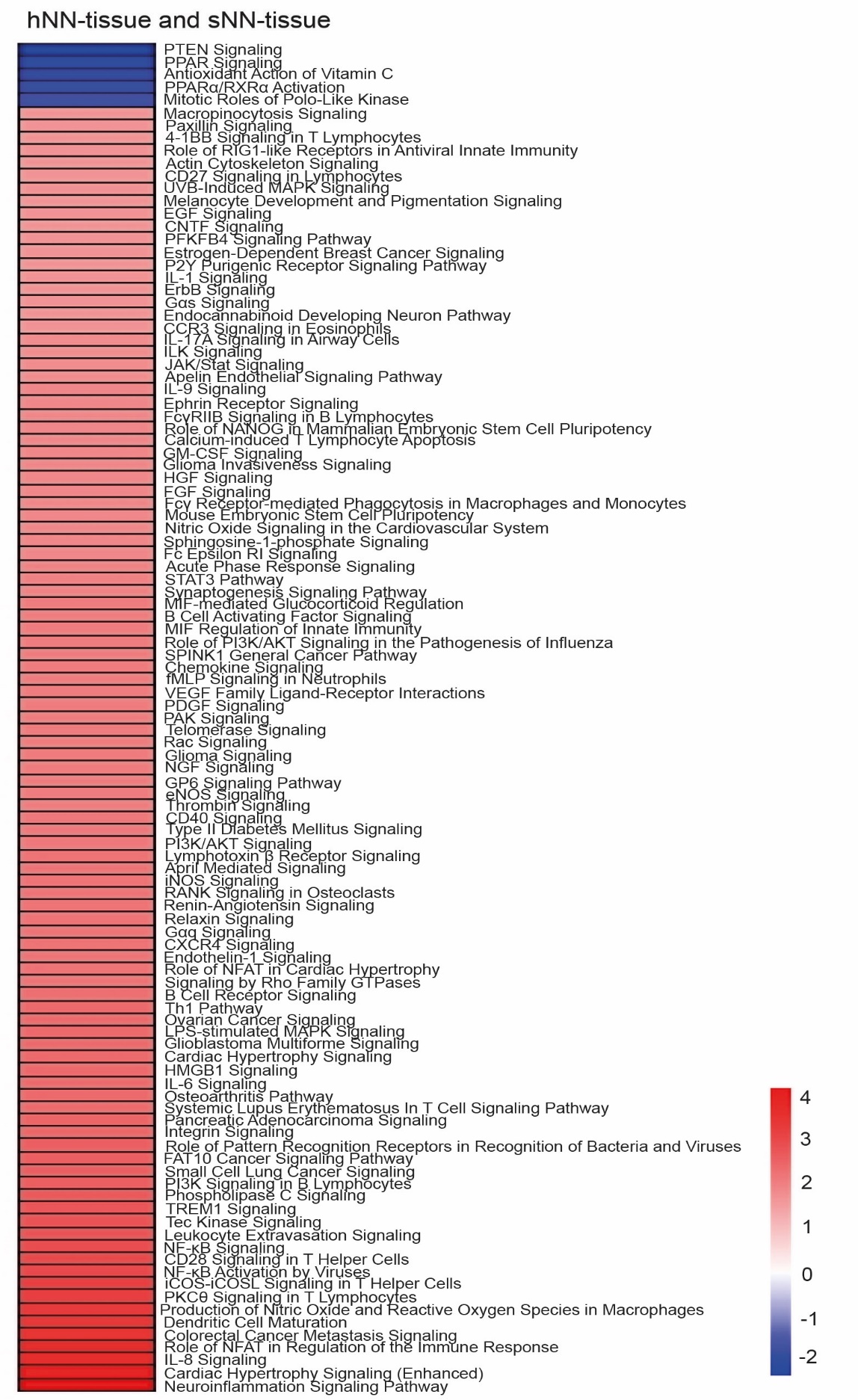
**

**S3 Fig.** Heatmap of differentially activated pathways when comparing non-neoplastic squamous tissue from HL survivors (hNN-tissue) versus non-neoplastic squamous tissue from sporadic ESCC cases (sNN-tissue).

**
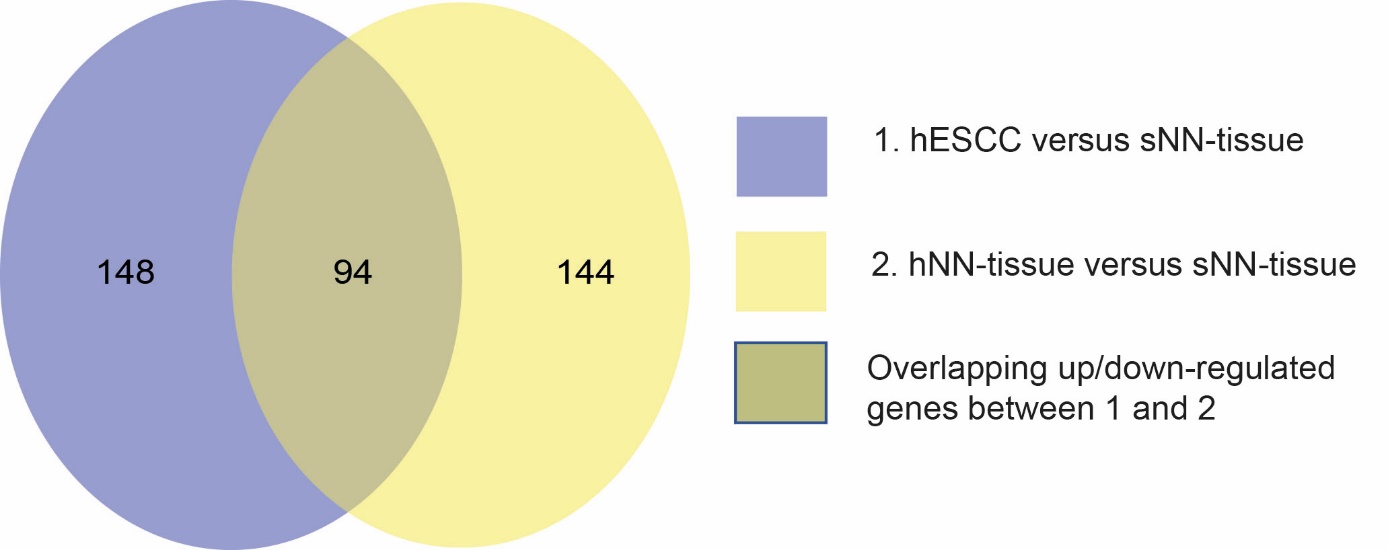
**

**S4 Fig.** Venn diagram showing common and different genes when comparing the gene-lists resulting from differentially expression analyses comparing
1. Non-neoplastic tissue from HL survivors (hNN-tissue) versus non-neoplastic tissue from sporadic ESCC (sNN-tissue).
2. ESCC in HL survivors (hESCC) versus non-neoplastic tissue from sporadic ESCC (sNN-tissue).
The overlapping 94 genes are visualized in Fig 6.

**References**

1. Abnet, C.C., M. Arnold, and W.Q. Wei, *Epidemiology of Esophageal Squamous Cell Carcinoma.* Gastroenterology, 2018. **154**(2): p. 360-373.

2. Cancer Genome Atlas Research, N., et al., *Integrated genomic characterization of oesophageal carcinoma.* Nature, 2017. **541**(7636): p. 169-175.

3. *NanostringNorm. Available:* [*https://CRAN.R-project.org/package=NanoStringNorm*](https://CRAN.R-project.org/package=NanoStringNorm) *[Accessed August 2019].*

4. Rigter, L.S., et al., *Double somatic mutations in mismatch repair genes are frequent in colorectal cancer after Hodgkin's lymphoma treatment.* Gut, 2018. **67**(3): p. 447-455.
